# Multidrug-Resistant Profiles in Non-Small Cell Lung Carcinoma Patient-Derived Cells: Implications for Personalized Approaches with Tyrosine Kinase Inhibitors

**DOI:** 10.3390/cancers16111984

**Published:** 2024-05-23

**Authors:** Jelena Dinić, Miodrag Dragoj, Sofija Jovanović Stojanov, Ana Stepanović, Ema Lupšić, Milica Pajović, Thomas Mohr, Sofija Glumac, Dragana Marić, Maja Ercegovac, Ana Podolski-Renić, Milica Pešić

**Affiliations:** 1 Department of Neurobiology, Institute for Biological Research “Siniša Stanković”—National Institute of the Republic of Serbia, University of Belgrade, Bulevar Despota Stefana 142, 11108 Belgrade, Serbia; jelena.dinic@ibiss.bg.ac.rs (J.D.); miodrag.dragoj@ibiss.bg.ac.rs (M.D.); sofija.jovanovic@ibiss.bg.ac.rs (S.J.S.); ana.kostic@ibiss.bg.ac.rs (A.S.); ema.lupsic@ibiss.bg.ac.rs (E.L.); milica.pajovic@ibiss.bg.ac.rs (M.P.); ana.podolski@ibiss.bg.ac.rs (A.P.-R.); 2 Center for Cancer Research, Comprehensive Cancer Center, Medical University of Vienna, Borschkegasse 8a, 1090 Vienna, Austria; thomas.mohr@mohrkeg.co.at; 3 Institute of Pathology, School of Medicine, University of Belgrade, Dr. Subotića 1, 11000 Belgrade, Serbia; sofija.glumac@med.bg.ac.rs; 4 School of Medicine, University of Belgrade, Dr. Subotića 8, 11000 Belgrade, Serbia; dragana.maric@med.bg.ac.rs (D.M.); maja.ercegovac@med.bg.ac.rs (M.E.); 5 Clinic for Pulmonology, University Clinical Center of Serbia, Dr. Koste Todorovića 26, 11000 Belgrade, Serbia

**Keywords:** lung cancer, NSCLC, multidrug resistance, ABCB1, ABCC1, ABCG2, tyrosine kinase inhibitors, targeted therapy, primary cell cultures, genomics

## Abstract

**Simple Summary:**

This research investigates the challenge of drug resistance in non-small cell lung carcinoma (NSCLC) and how certain drugs, namely, tyrosine kinase inhibitors (TKIs), can induce multidrug resistance (MDR). This study aims to understand how patient-derived NSCLC cells respond to different TKIs and how genetic variations in patients may influence this response. The analysis of the efficacy of TKIs and their influence on the expression of specific markers associated with MDR shows that they elicit a different response in different NSCLC cells. Genetic alterations in signaling pathways associated with drug resistance likely contribute to the differential responses to TKIs. These findings underscore the importance of considering individual genetic profiles and performing thorough sensitivity testing to develop effective treatment strategies, particularly in the early stages of NSCLC, and highlight the potential for personalized cancer therapies.

**Abstract:**

The impact of tyrosine kinase inhibitors (TKIs) on multidrug resistance (MDR) in non-small cell lung carcinoma (NSCLC) is a critical aspect of cancer therapy. While TKIs effectively target specific signaling pathways of cancer cells, they can also act as substrates for ABC transporters, potentially triggering MDR. The aim of our study was to evaluate the response of 17 patient-derived NSCLC cultures to 10 commonly prescribed TKIs and to correlate these responses with patient mutational profiles. Using an ex vivo immunofluorescence assay, we analyzed the expression of the MDR markers ABCB1, ABCC1, and ABCG2, and correlated these data with the genetic profiles of patients for a functional diagnostic approach. NSCLC cultures responded differently to TKIs, with erlotinib showing good efficacy regardless of mutation burden or EGFR status. However, the modulation of MDR mechanisms by erlotinib, such as increased ABCG2 expression, highlights the challenges associated with erlotinib treatment. Other TKIs showed limited efficacy, highlighting the variability of response in NSCLC. Genetic alterations in signaling pathways associated with drug resistance and sensitivity, including TP53 mutations, likely contributed to the variable responses to TKIs. The relationships between ABC transporter expression, gene alterations, and response to TKIs did not show consistent patterns. Our results suggest that in addition to mutational status, performing functional sensitivity screening is critical for identifying appropriate treatment strategies with TKIs. These results underscore the importance of considering drug sensitivity, off-target effects, MDR risks, and patient-specific genetic profiles when optimizing NSCLC treatment and highlight the potential for personalized approaches, especially in early stages.

## 1. Introduction

Non-small cell lung carcinoma (NSCLC) represents a significant health burden worldwide and accounts for the majority of all lung cancer cases [1]. Despite advances in diagnosis and treatment modalities, NSCLC remains a major challenge as it tends to develop multidrug resistance (MDR), which is a major obstacle to effective therapy. Intrinsic MDR occurs when cancer cells are inherently resistant to chemotherapy due to genetic mutations or alterations, making standard treatments less effective [2]. In contrast, acquired MDR develops during treatment as cancer cells adapt to repeated exposure to anticancer drugs, often leading to treatment failure and disease recurrence. In this context, ATP-binding cassette (ABC) transporters have attracted considerable attention due to their role in the development of MDR in NSCLC. ABC transporters are a family of membrane-bound proteins that play a critical role in the cellular efflux of various agents, including drugs and xenobiotics [3], which may limit the efficacy of chemotherapy and targeted therapies. Of the ABC transporters, ABCB1, ABCC1, and ABCG2 are involved in drug resistance in a variety of malignancies. ABCB1, also known as P-glycoprotein, was the first ABC transporter identified and is strongly associated with MDR in several cancer types, including NSCLC, breast, colorectal, and prostate cancer [4]. Similarly, high levels of ABCG2, known as breast cancer resistance protein (BCRP), have been associated with MDR in NSCLC, melanoma, breast, colorectal, ovarian, and gastric cancers [5]. In addition to NSCLC, the overexpression of ABCC1, also known as multidrug resistance protein 1 (MRP1), has been associated with breast, ovarian, prostate, colorectal, and pancreatic cancers [2]. Understanding the complex role of ABCB1, ABCC1, and ABCG2 in acquired resistance is critical for improving strategies to overcome MDR, increasing treatment efficacy, and ultimately improving outcomes for cancer patients.

In the search for more effective treatments, tyrosine kinase inhibitors (TKIs) have emerged as promising agents, particularly for patients with specific genetic mutations that drive their tumors. This group of targeted therapeutics includes agents such as afatinib, alectinib, ceritinib, crizotinib, dabrafenib, erlotinib, gefitinib, nintedanib, osimertinib, and trametinib, all of which aim to block specific signaling pathways responsible for tumor growth and progression [6]. These TKIs have transformed the treatment of NSCLC, particularly in patients with sensitizing mutations such as epidermal growth factor receptor (EGFR) mutations, anaplastic lymphoma kinase (ALK) rearrangements, and ROS1 rearrangements [7,8]. Drugs such as gefitinib and erlotinib, which target EGFR mutations, have shown exceptional success in improving progression-free survival and overall response rates [9,10]. Similarly, crizotinib, alectinib, and ceritinib have improved outcomes in NSCLC patients with ALK or ROS1 rearrangements [11,12].

Although TKIs represent a significant advance in the treatment of NSCLC compared to classical chemotherapy, MDR remains a challenge [13]. Ongoing research is aimed at understanding the mechanisms of TKI resistance and developing innovative strategies. The study of primary and acquired resistance to TKIs, particularly in EGFR-mutated NSCLC, remains a critical area of research to improve patient outcomes [14]. The combination of TKIs with other agents is becoming increasingly important, and next-generation TKIs such as osimertinib for EGFR T790M mutations offer significant advances in the treatment of resistant NSCLC [15]. While ALK and EGFR TKIs are used to treat NSCLC patients with specific genetic alterations, they can also cause a wide range of responses in patients with identical genetic mutations [16]. For example, certain patients with EGFR and ALK mutations may not respond to TKIs, while others may show either a partial response or complete remission [16,17]. These differences in treatment outcomes are not fully understood and may depend on several factors, including specific mutations, the presence of concurrent genetic alterations, or differences in the tumor microenvironment [16]. In addition, some rare EGFR mutations may also influence the response to TKIs. For example, mutations such as p.Glu719X and p.Ser768Gln have been associated with response to second-generation TKIs such as afatinib, while other mutations may predict primary resistance or decreased sensitivity to first- and second-generation EGFR TKIs [18,19].

Whole-exome sequencing (WES) can be part of a functional diagnostic approach for NSCLC by providing a comprehensive molecular profile of the patient’s tumor. This information can be used to identify the genetic basis of MDR, allowing clinicians to select the most appropriate targeted therapy for their patients [20]. The data obtained with WES can significantly advance precision medicine, including the identification of rare genetic alterations that are undetectable with routine testing [21].

In this study, we examined the expression of MDR markers, specifically ABCB1, ABCC1, and ABCG2, in 17 patient-derived NSCLC cultures after exposure to 10 commonly prescribed TKIs for the treatment of NSCLC. The expression of MDR markers was determined using an ex vivo immunofluorescence assay that can effectively distinguish between the MDR profiles of cancer and non-cancer stromal cells in mixed cell cultures. We correlated the response of each culture with the genetic profiles of the corresponding patients as part of a functional diagnostics approach.

## 2. Materials and Methods

### 2.1. NSCLC Tissue Samples and Establishment of Primary Cultures

The tissue samples from NSCLC patients were obtained from the Clinic for Thoracic Surgery at the University Clinical Center of Serbia after obtaining informed consent from the patients and approval from the Ethics Committee of the University Clinical Center of Serbia (approval reference number 623/4). The NSCLC patients who provided tissue samples for the study were randomly selected. The samples were collected during surgery, and histopathologic analysis determined NSCLC diagnosis, histologic grade, stage, necrosis, and lymph node invasion status. An EGFR L858R mutation was detected in the TR64 sample [22]. The clinical parameters of the patients with NSCLC are listed in Table S1. After surgical removal, the samples were placed in a sterile tube containing antibiotic-antimycotic solution (Sigma-Aldrich Chemie GmbH, Taufkirchen, Germany) and immediately transferred to the research laboratory for further processing.

To establish primary cultures, the tissue was manually minced with a surgical blade in a sterile Petri dish upon arrival at the laboratory. The samples were cut into 3–5 mm pieces and dissociated using the Tumor Dissociation Kit (Miltenyi Biotec, Bergisch Gladbach, Germany) according to the manufacturer’s instructions. The tissue pieces were incubated in an orbital shaker (KS 4000 ic control, IKA, Königswinter, Germany) at 37 °C and 300 rpm for 90 min. After incubation, the dissociated tissue was placed in DMEM/Ham’s F12 (1:3) growth medium supplemented with 5% fetal bovine serum (Corning, NY, USA), antibiotic-antimycotic solution, 4 µg/mL hydrocortisone (Sigma-Aldrich Chemie GmbH, Taufkirchen, Germany), 1 µg/mL insulin (Sigma-Aldrich Chemie GmbH, Taufkirchen, Germany), 10 ng/mL epidermal growth factor (BioLegend, San Diego, CA, USA), and 24 µg/mL adenine (Sigma-Aldrich Chemie GmbH, Taufkirchen, Germany). The growth media DMEM and Ham’s F12 were purchased from Sigma-Aldrich Chemie GmbH (Taufkirchen, Germany). The dissociated tissue was cultured in T-25 cell culture flasks until cell attachment was observed before the medium was replaced. Samples in which no cell attachment was observed within 7 days were discarded. Successfully established primary NSCLC cultures were maintained at 37 °C in a humidified atmosphere with 5% CO_2_ and grown to confluence prior to further experiments.

Tumor tissue and corresponding normal tissue samples from NSCLC patients were snap-frozen in liquid nitrogen for later use in DNA isolation for whole-exome sequencing. The tissue samples were stored in liquid nitrogen until use.

### 2.2. Drugs and Treatments

Afatinib, crizotinib, erlotinib, gefitinib, and nintedanib were purchased from Sigma-Aldrich Chemie GmbH (Taufkirchen, Germany). Alectinib, ceritinib, dabrafenib, osimertinib, and trametinib were purchased from Selleckchem (Houston, TX, USA). All compounds were dissolved in DMSO and stored at −20 °C. Prior to treatment, the stock solutions were freshly diluted in sterile water. The drugs were applied at clinically relevant concentrations. The maximum concentration reached in human plasma to which the patient is exposed during therapy (*C*_max_) was set as the upper limit, and four lower concentrations were also used [23]: afatinib (10, 20, 30, 40, and 50 nM); alectinib (0.5, 0.75, 1, 1.25, and 1.5 µM); ceritinib (0.5, 0.75, 1, 1.25, and 1.5 µM); crizotinib (100, 250, 500, 750, and 1000 nM); dabrafenib (1, 2, 3, 4, and 5 µM); erlotinib (0.5, 1, 2, 3, and 4 µM); gefitinib (50, 100, 200, 300, and 400 nM); nintedanib (2.5, 5, 10, 15, and 20 µM); osimertinib (50, 75, 100, 125, and 150 nM); and trametinib (5, 10, 15, 20, and 25 nM).

Patient-derived cells were seeded in black clear-bottom 384-well cell culture microplates (Thermo Fisher Scientific, Waltham, MA, USA) at a density of 1000 cells per well in 50 µL of cell growth medium. The drugs were administered 72 h after seeding and the treatment lasted for 7 days.

### 2.3. Immunofluorescence Assay

The immunofluorescence assay was optimized to quantify MDR markers and distinguish cancer cells from stromal cells using a cytokeratin 8/18 (CK8/18) antibody mixture as previously described [22]. CK8/18-positive cells were considered as cancer cells, while CK8/18-negative cells were considered as stromal cells. Cells were fixed in 4% paraformaldehyde for 20 min at RT and washed using the Wellwash™ Versa microplate washer (Thermo Fisher Scientific, Waltham, MA, USA). The cells were then blocked with 2% bovine serum albumin (BSA) in PBS for 1 h at RT. The cells were then incubated overnight at 4 °C with a primary rabbit CK8/18 antibody cocktail (clone SU0338, #MA5-32118, Thermo Fisher Scientific, Waltham, MA, USA) and primary mouse antibodies against ABCB1 (clone C219, #MA1-26528, Thermo Fisher Scientific, Waltham, MA, USA), ABCC1 (clone IU5C1, #MA5-16079, Thermo Fisher Scientific, Waltham, MA, USA), or ABCG2 (clone 1H2, #ab130244, Abcam, Cambridge, UK). The cells were then incubated with secondary Alexa Fluor 555 goat anti-mouse antibody (#A-21422, Thermo Fisher Scientific, Waltham, MA, USA) and secondary Alexa Fluor 488 goat anti-rabbit antibody (#A-11008, Thermo Fisher Scientific, Waltham, MA, USA) at RT for 2 h under photoprotective conditions. The cell nuclei were counterstained with 1 µg/mL Hoechst 33342 at RT for 2 h. The cells were stored at 4 °C in the dark prior to imaging.

Fluorescently labeled cells were imaged using the ImageXpress^®^ Pico Automated Cell Imaging System (Molecular Devices^®^, San Jose, CA, USA) with a 4x objective after determining the appropriate exposure time for each illumination filter. The data analysis of the obtained images was performed using CellReporterXpress^®^ software v. 2.8.2.669 (Molecular Devices^®^, San Jose, CA, USA). The Cell Scoring Analysis Protocol was used to assess the cytotoxicity of drugs as previously described [22]. To determine the expression of MDR markers, the Multi-Wavelength Cell Scoring Analysis Protocol was used as previously described [22].

### 2.4. Whole-Exome Sequencing and Variant Calling

DNA extraction from tumor and corresponding normal tissue samples from NSCLC patients was performed using the QIAamp Fast DNA Tissue Kit (Qiagen, Hilden, Germany) according to the manufacturer’s protocol. DNA was quantified using a NanoPhotometer^®^ N60 (IMPLEN, Munich, Germany), and its quality and integrity was checked by means of electrophoretic separation on a 0.8% agarose gel. All DNA samples had the required concentration (≥20 ng/μL) and were sent in a final volume of 20 μL to Novogene Co, Ltd. (Cambridge, UK) for whole-exome sequencing using the NovaSeq 6000 instrument (Illumina, San Diego, CA, USA). The SureSelect Human AII Exon V6 capture baits Kit (Agilent Technologies, Inc., Santa Clara, CA, USA) was used for DNA library preparation.

Sequence data were analyzed using a custom bioinformatics pipeline in Novogene, which aligns the sequence data to the human genome (GRCh37) to perform variant calling and annotation. Briefly, fastp (version 0.23.1) was used to assess the sequence quality of the resulting paired-end 150-nucleotide reads [24]. Reads were mapped to the human reference genome GRCh37 using BWA software (version 0.7.8-r455) [25] and duplicates were tagged using the Picard tool (version 2.6.0). For the detection of somatic SNPs/InDels, Varscan2 (version v2.4.3) was used [26]. Variant annotation was performed using the Variant Effect Predictor (VEP) tool (ensembl-vep-release-105). VEP can utilize a variety of annotation sources to retrieve the transcript models used to predict the consequence types [27]. The main databases used include RefSeq, dbSNP, COSMIC, ClinVar, 1000 Genomes, NHLBI-ESP, genomAD, SIFT, PolyPhen, and HGMD-PUBLIC. All results were integrated and analyzed with statistical methods in R/Bioconductor [28]. For further analysis, we selected only non-synonymous variants corresponding to the following functional classification: Frame_Shift_Del, Frame_Shift_Ins, In_Frame_Del, In_Frame_Ins, Missense_Mutation, Nonsense_Mutation, Nonstop_Mutation, and Splice_Site. We used the maftools R package to analyze and visualize the mutational landscapes of tumor samples [29].

### 2.5. Variant Prioritization and Pathway Analysis

Variants were filtered against a cancer-related gene panel from the databases of the Catalog Of Somatic Mutations In Cancer (COSMIC) Cancer Gene Census [30]. Further, the variants were scored using the SIFT and PolyPhen2 tools to predict the effects of the mutation on the respective protein. Variants that were found to have deleterious effects were selected for further analysis. Variants with a gnomAD allele frequency of less than 0.01 were selected for further analysis, focusing on rare variants that are less frequently observed in the general population and therefore more likely to be pathogenic. Variants were also prioritized based on their annotations in the ClinVar database [31]. Annotations that were considered significant for this analysis included “pathogenic”, “likely_pathogenic”, “pathogenic/likely_pathogenic”, and “drug_response”. After applying the above criteria, the prioritized variants from different filters were merged into a single set to ensure the exclusion of duplicates.

To analyze the pathways, Bioconductor’s clusterProfiler [32] package was used to perform a functional enrichment analysis using Fisher’s exact test. The enrichKEGG function was used to identify the biological pathways involved. This method enabled the comprehensive identification and analysis of significant pathways in the dataset. The analysis included a Bonferroni correction to adjust for multiple comparisons, with the significance threshold set at an adjusted *p*-value of 0.05. Non-relevant pathways, such as those related to cancer in general and other diseases such as infectious diseases and addiction, were excluded from our analysis.

### 2.6. Statistical Analysis

The statistical analysis of the immunofluorescence assay data was performed using GraphPad Prism software version 8.0.2 (San Diego, CA, USA). Data were subjected to two-way analysis of variance (ANOVA), followed by Dunnett’s test for multiple comparisons. Statistical significance was considered given if the *p*-values were less than 0.05.

Spearman’s rank correlation coefficient was utilized to evaluate the non-parametric statistical dependence among variables, including stage, sensitivity to TKIs, intrinsic resistance, mutational burden, and pathogenic mutations. The data were transformed into ranks, with stages ranging from 1 to 4, sensitivity to TKIs from 1 to 6 (number of TKIs to which patient sample was sensitive), intrinsic resistance from 0 to 3 (number of ABC transporters with high expression), the level of mutational burden from 0 to 4, and the presence of pathogenic mutations from 0 to 2. The differences between the ranks of the paired observations were then examined. The resulting correlation coefficient, which ranges from −1 to 1, indicates a perfect positive relationship at 1, a perfect negative relationship at −1, and no relationship at 0. Spearman r and p values were computed using GraphPad Prism software version 8.0.2 (San Diego, CA, USA).

## 3. Results

### 3.1. Assessment of Sensitivity of Patient-Derived NSCLC Cultures to TKIs

**Figure 1:**
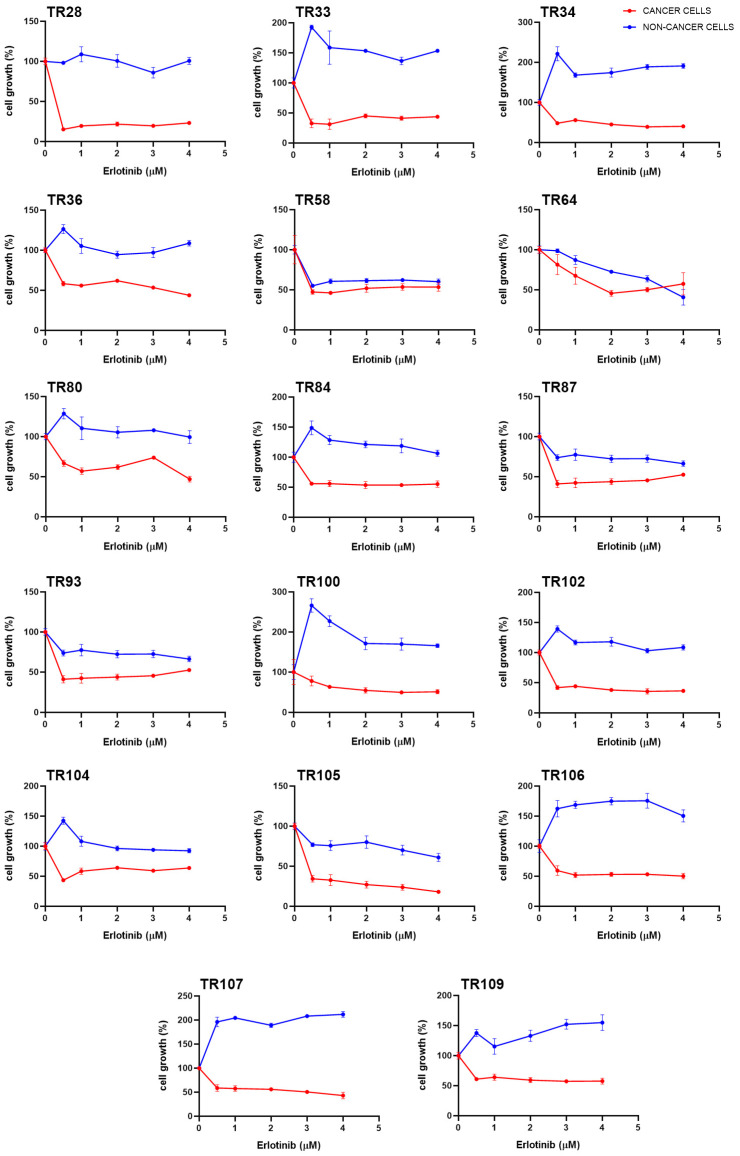
Effect of erlotinib on cell growth in patient-derived NSCLC cultures. The CK8/18 antibody was used to differentiate between cancer cells and non-cancer cells in a mixed culture. The NSCLC cultures were treated for 7 days. Values are expressed as mean ± SEM (*n* = 4).

**Figure 2:**
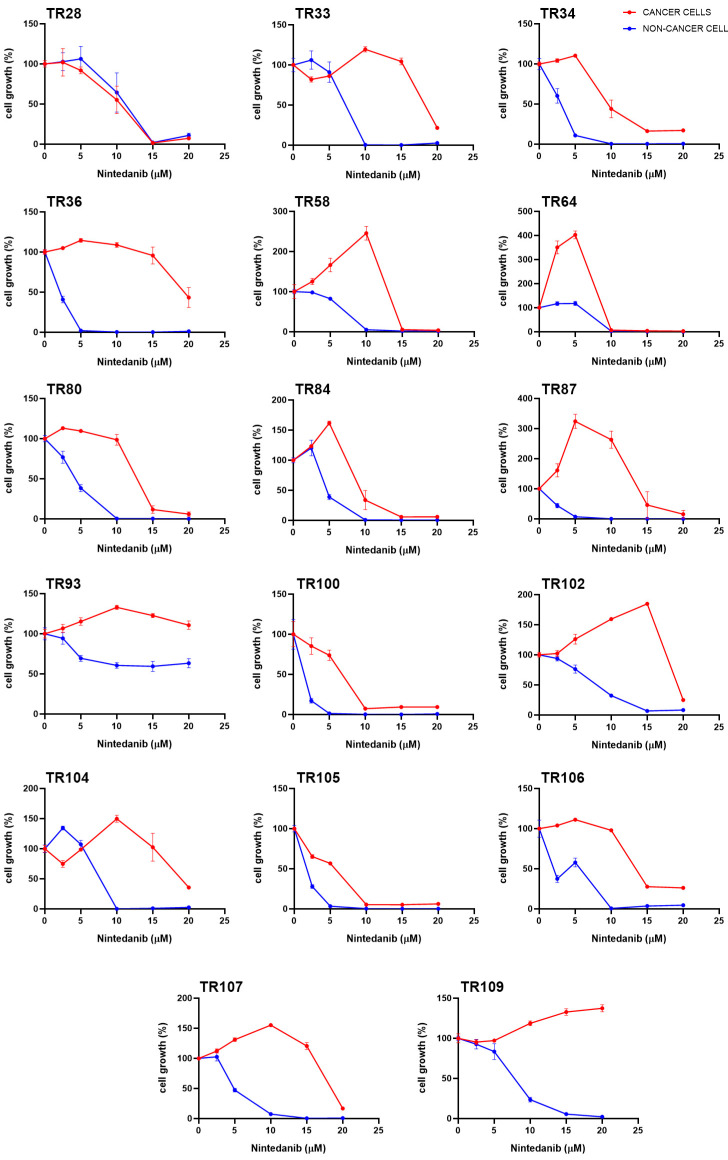
Effect of nintedanib on cell growth in patient-derived NSCLC cultures. The CK8/18 antibody was used to differentiate between cancer cells and non-cancer cells in a mixed culture. The NSCLC cultures were treated for 7 days. Values are expressed as mean ± SEM (*n* = 4).

The primary NSCLC cultures were treated with afatinib, alectinib, ceritinib, crizotinib, dabrafenib, erlotinib, gefitinib, nintedanib, osimertinib, and trametinib. The IC_50_ values, which indicate the concentration required to inhibit cell growth by 50%, for the different TKIs showed diverse response patterns in different cultures (Table 1). Among the drugs tested, erlotinib showed notable efficacy, with positive responses observed in CK8/18+ cancer cells in all cultures tested. In addition, erlotinib selectively inhibited the proliferation of CK8/18+ cancer cells in NSCLC cultures compared to CK8/18− stromal cells. The response to erlotinib varied between cancer cells in different NSCLC cultures, as indicated by different IC_50_ values. The effect of erlotinib on cell growth in patient-derived NSCLC cultures is shown in Figure 1. In most cultures of NSCLC (CK8/18+ cancer cells), a noticeable decrease in cell growth was observed at lower concentrations of erlotinib. There was no clear dose–response effect of erlotinib as a plateau was evident in almost all NSCLC cultures (CK8/18+ cancer cells). On the other hand, nintedanib showed a dual effect on cancer cells, promoting cancer cell growth at lower concentrations (below 10 µM) and inducing 90% cell death in most cultures at higher concentrations (above 15 µM). The effect of nintedanib on cell growth in patient-derived NSCLC cultures is shown in Figure 2.

**Figure 3:**
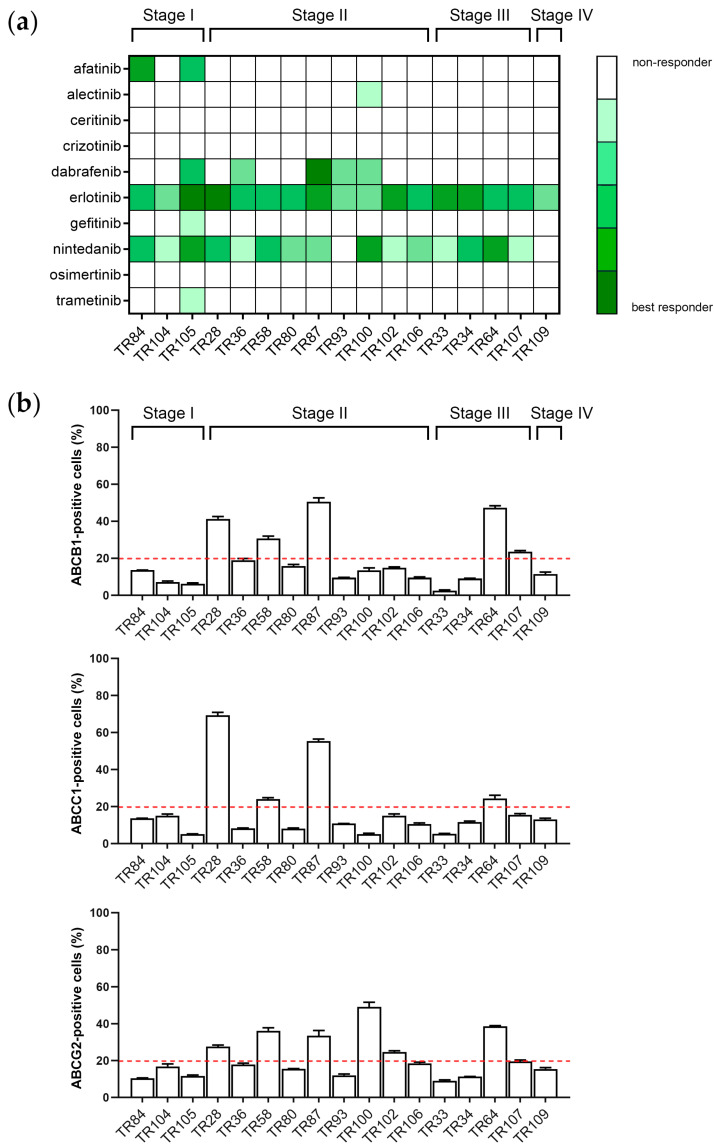
Response of cancer cells in patient-derived NSCLC cultures to tyrosine kinase inhibitors. (**a**) The NSCLC cultures were categorized into six groups according to the response of the CK8/18+ cells to the TKIs, from “best responder” (strong response, IC50 closest to the lowest TKI concentration) to “non-responder”. (**b**) The intrinsic expression of ABC transporters ABCB1, ABCC1, and ABCG2 in NSCLC cultures. The graphs show the percentage of cells positive for the ABCB1, ABCC1, and ABCG2 antibodies. NSCLC cultures were considered inherently resistant if at least 20% of the cells were positive for at least one ABC transporter (red line). Data are presented as mean ± SEM (*n* = 4).

In contrast to erlotinib and nintedanib, other TKIs were generally ineffective against cancer cells in NSCLC cultures. Figure 3a summarizes the response of CK8/18+ cells in the tested NSCLC cultures to 10 TKIs. Cancer cells from some patients with stage I or II NSCLC (e.g., TR36, TR84, TR87, and TR100) were sensitive to several TKIs, with some samples (e.g., TR105) showing sensitivity to a broad range of inhibitors. Cancer cells from stage III or IV patients (e.g., TR33, TR34, TR64, and TR107) generally showed a more limited response, restricted to erlotinib and nintedanib. The intrinsic expression of ABC transporters ABCB1, ABCC1, and ABCG2 in CK8/18+ cells in NSCLC cultures is shown in Figure 3b. NSCLC patient-derived cells were categorized into sensitive and resistant groups based on the initial expression of ABC markers (before treatment to TKIs) and response to TKIs. Sensitive cultures were defined as those that responded to ≥2 TKIs and in which less than 20% of the cells inherently expressed all three ABC transporters, while resistant cultures were defined as those that responded to ≤2 TKIs and in which at least 20% of the cells inherently expressed at least one ABC transporter. We found that among sensitive NSCLC cells, i.e., those responding to ≥2 TKIs (TR84, TR105, TR36, TR87, and TR100), not all had less than 20% of CK8/18+ cells that intrinsically expressed ABC transporters (TR87 and TR100) (Figure 3b). NSCLC cells that were considered inherently resistant, with at least 20% of CK8/18+ cells positive for at least one ABC transporter, were TR28, TR58, TR102, TR64, and TR107, and they were in line with the applied categorization. However, samples TR104, TR80, TR93, TR106, TR33, TR34, and TR109, that were sensitive to only two TKIs, had less than 20% of positive cells for all ABC transporters.

The IC_50_ values for non-cancer CK8/18− cell populations within the primary cultures also showed different responses to the various targeted drugs (Table 1). In the majority of cultures tested, CK8/18− cells showed no sensitivity to TKIs. Dabrafenib was effective in both CK8/18+ and CK8/18− cells in several NSCLC cultures (TR36, TR87, and TR100), while crizotinib showed a preference for non-cancer cell populations in five NSCLC cultures (TR34, TR36, TR80, TR87, and TR100) but was ineffective in cancer cells. Nintedanib showed a strong response in the majority of CK8/18− populations, while the other TKIs were generally ineffective in stromal cells.

### 3.2. Assessment of MDR Marker Expression in Patient-Derived NSCLC Cultures after Treatment with TKIs

Next, we examined the expression of MDR markers, specifically ABCB1, ABCC1, and ABCG2, in NSCLC cultures after treatment with afatinib, alectinib, ceritinib, crizotinib, dabrafenib, erlotinib, gefitinib, nintedanib, osimertinib, and trametinib.

Table 2 shows CK8/18+ NSCLC cultures with increased ABCB1, ABCC1, and ABCG2 expression after treatment with TKIs. A threshold of at least 20% increase in MDR marker expression was considered biologically relevant. The TKIs showed different effects on the expression of MDR markers in CK8/18+ cells, with afatinib and alectinib each affecting one primary culture (TR87 and TR107, respectively). In two cultures, the expression of MDR markers was increased after treatment with ceritinib (TR87 and TR107), dabrafenib (TR80 and TR107), and gefitinib (TR84 and TR87). The expression of MDR markers in these cultures was increased regardless of their ineffectiveness in terms of cell growth. Crizotinib, osimertinib, and trametinib showed no effect on the expression of MDR markers in all cultures.

Erlotinib, on the other hand, increased ABCG2 levels in 5 cultures (TR28, TR64, TR102, TR105, and TR107) and increased ABCB1 (TR28 and TR87) and ABCC1 expression (TR28 and TR64) in 2 of 17 cultures. The expression of MDR markers was increased at erlotinib concentrations that were effective in inhibiting cell growth. In contrast, nintedanib, which induced significant cell death at higher concentrations, enriched the NSCLC cultures with CK8/18+ cells with a high expression of ABCB1, ABCC1, and ABCG2. Specifically, increased ABCG2 expression was observed in 15 of 17 cultures and increased ABCB1 and ABCC1 expression was observed in 8 of 17 cultures. It is also worth noting that while nintedanib showed no efficacy in inhibiting cell growth in TR93, an increase in the expression of the ABCB1, ABCC1, and ABCG2 markers was observed. The TKIs had no effect on the expression of MDR markers in stromal cells.

**Figure 4:**
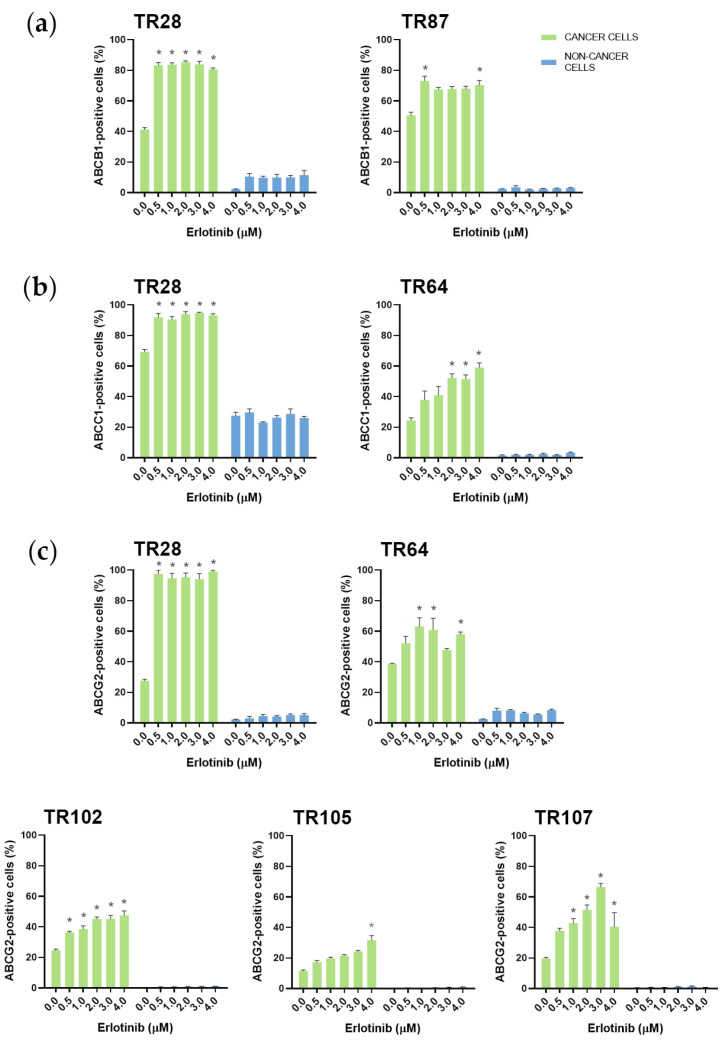
Patient-derived NSCLC cultures with increased expression of MDR markers after treatment with erlotinib. The CK8/18 antibody was used to differentiate between cancer cells and non-cancer cells in a mixed culture. The graphs show the percentage of cells positive for antibodies ABCB1 (**a**), ABCC1 (**b**) and ABCG2 (**c**) for each experimental condition. Data are presented as mean ± SEM (*n* = 4). Statistically significant differences between the control and treated groups showing an increase of at least 20% in ABCB1, ABCC1 and ABCG2 expression are indicated by * (*p* < 0.05).

**Figure 5:**
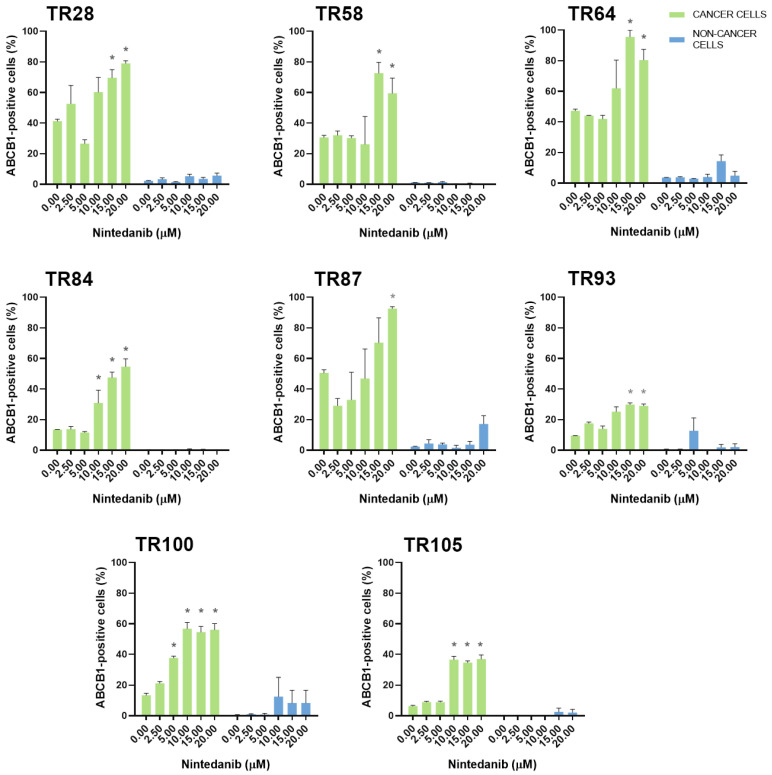
Patient-derived NSCLC cultures with an increased expression of ABCB1 after treatment with nintedanib. The CK8/18 antibody was used to differentiate between cancer cells and non-cancer cells in a mixed culture. The graphs show the percentage of cells positive for the ABCB1 antibody for each experimental condition. Data are presented as mean ± SEM (*n* = 4). Statistically significant differences between the control and treated groups showing an increase in ABCB1 expression of at least 20% are indicated by * (*p* < 0.05).

**Figure 6:**
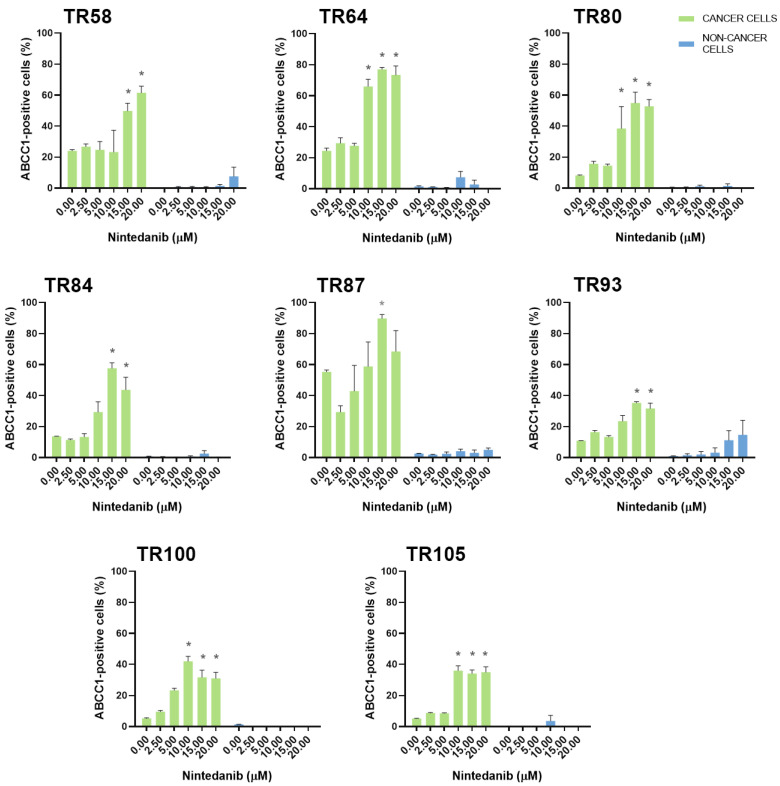
Patient-derived NSCLC cultures with an increased expression of ABCC1 after treatment with nintedanib. The CK8/18 antibody was used to differentiate between cancer cells and non-cancer cells in a mixed culture. The graphs show the percentage of cells positive for the ABCC1 antibody for each experimental condition. Data are presented as mean ± SEM (*n* = 4). Statistically significant differences between the control and treated groups showing an increase in ABCC1 expression of at least 20% are indicated by * (*p* < 0.05).

**Figure 7:**
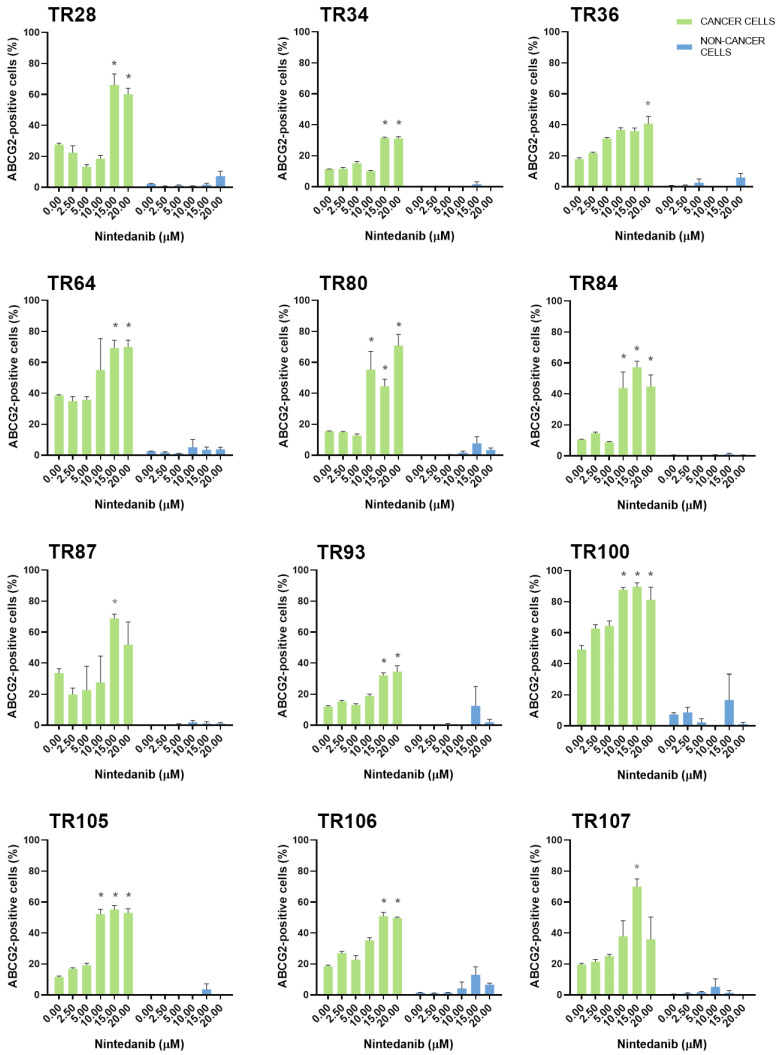
Patient-derived NSCLC cultures with an increased expression of ABCG2 after treatment with nintedanib. The CK8/18 antibody was used to differentiate between cancer cells and non-cancer cells in a mixed culture. The graphs show the percentage of cells positive for the ABCG2 antibody for each experimental condition. Data are presented as mean ± SEM (*n* = 4). Statistically significant differences between the control and treated groups showing an increase in ABCG2 expression of at least 20% are indicated by * (*p* < 0.05).

The increase in ABCB1, ABCC1, and ABCG2 expression in NSCLC cultures after treatment with five increasing concentrations of erlotinib is shown in Figure 4. The increase in ABCB1, ABCC1, and ABCG2 expression in NSCLC cultures after treatment with five increasing concentrations of nintedanib is shown in Figure 5, Figure 6, and Figure 7, respectively. The majority of stromal cells in NSCLC cultures (more than 95%) were negative for ABCB1, ABCC1, and ABCG2. Following treatment with erlotinib or nintedanib, some stromal cells showed a slight increase in ABCB1 and ABCG2 expression after erlotinib treatment (in TR28 and TR64, respectively). In addition, some CK8/18− cells showed a slight increase in ABCB1 expression (in TR64, TR87, TR93, and TR100), ABCC1 expression (in TR58, TR64, and TR93) and ABCG2 expression (in TR28, TR36, TR80, TR93, TR100, and TR106) after nintedanib treatment. Nevertheless, these increases were not significant, with more than 90% of stromal cells not showing positivity for ABCB1, ABCC1, or ABCG2 upon treatment with erlotinib and nintedanib (Figure 4, Figure 5 and Figure 6). The expression of ABCC1 in TR28 was an exception, as non-cancer cells in this culture showed a high intrinsic expression of ABCC1. However, treatment with erlotinib increased the expression of this MDR marker only in cancer cells, while no effects on ABCC1 were observed in non-cancer cells.

### 3.3. Mutational Landscape of NSCLC Patients

**Figure 8:**
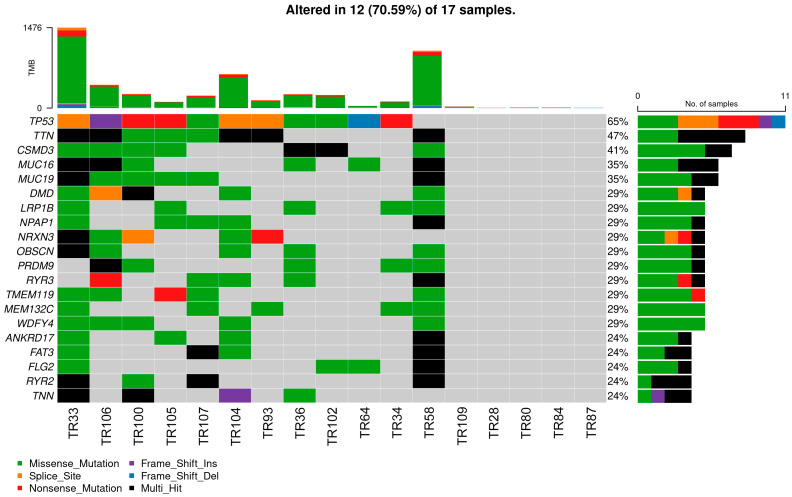
Oncoplot depicting the mutational landscape of NSCLC patients. Each column corresponds to an individual sample. Each row represents a specific gene. The color-coding indicates the mutation type. The top sidebar plots indicate the tumor mutation burden (TMB).

Whole-exome sequencing was performed on 17 NSCLC samples and identified 5044 non-synonymous mutations, including 4256 missense mutations, 214 frame-shift insertions/deletions, 64 in-frame insertions/deletions, 374 nonsense mutations, 6 non-stop mutations, 12 translation start site mutations, and 118 splice site mutations. The median number of variants per sample was 114, with considerable variation between samples (Supplementary Figure S1). We used an oncoplot to visualize the mutational landscape across the cohort (Figure 8). Notably, TP53 was the most frequently mutated gene, with alterations detected in 65% of patients. In addition, our analysis revealed considerable variability in tumor mutational burden (TMB) in the patient population. The oncoplot showed a broad spectrum of TMB values, indicating a heterogeneous mutational landscape in our cohort. In particular, samples TR33, TR58, TR104, and TR106 had the highest TMB, suggesting a more complex mutational profile. Conversely, samples TR28, TR80, TR84, and TR87 had the lowest TMB, indicating a comparatively stable genomic architecture.

### 3.4. Pathway Analysis

**Figure 9:**
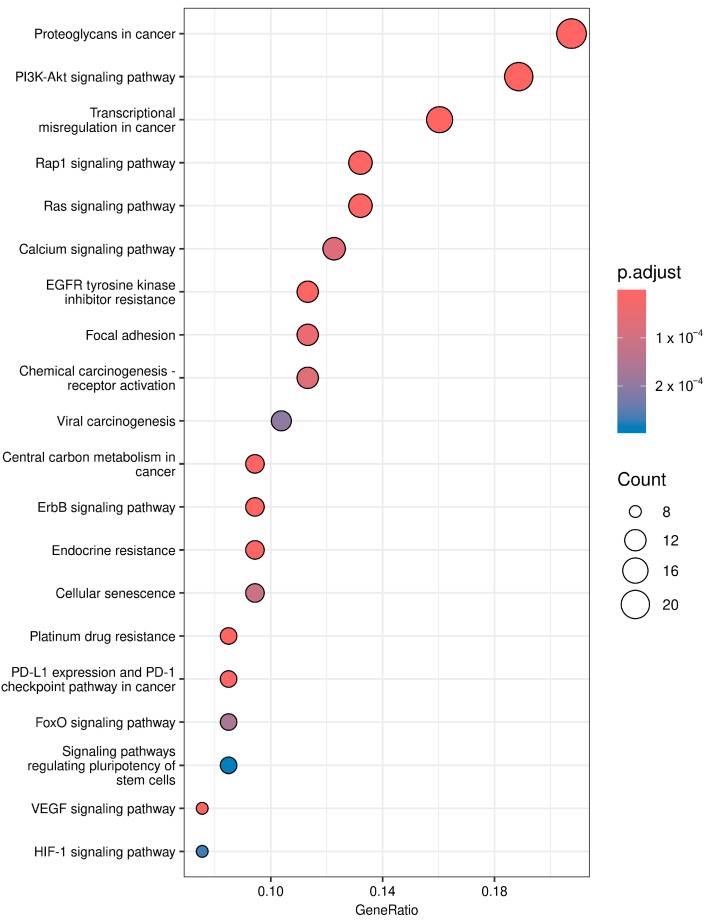
KEGG pathway enrichment analysis. The dot plot illustrates KEGG pathway enrichment among genes with mutations from WES data. The dot size reflects the number of genes per pathway. The color indicates the significance level (*p*-adjusted value).

To investigate which genes are mutated in the different pathway classes, the information from the Whole Exome Sequencing (WES) data was mapped to the KEGG pathways. Only rare variants (gnomAD allele frequency < 0.01) of genes that met the filter criteria using a cancer gene panel from COSMIC Cancer Gene Census, also chosen based on SIFT and PolyPhen2 scores and ClinVar annotations (e.g., “pathogenic”), were used for KEGG pathway analysis. The pathway analysis and functional annotation of these selected variants were performed using the R programming package clusterProfiler to ensure that our KEGG enrichment analysis focused exclusively on the most important genetic variations. The 20 most enriched pathways are shown in Figure 9.

The results from the KEGG pathways analysis are presented in Supplementary Table S2.

A clinical significance of detected mutations in prioritized genes identified in 20 KEGG pathways (Table S2) was found for several patients [31]. These mutations are mainly associated with the PI3K-Akt signaling pathway and Platinum drug resistance (Table S2).

TR105 (stage IB, squamous-cell carcinoma, 66-year-old male heavy smoker) has a TP53 non-sense mutation c.637C>T characterized as pathogenic with high impact on protein function and reported in Li–Fraumeni syndrome, as well as different cancer types. This patient showed the best response to TKIs in our cohort, responding to 6 out of 10 TKIs (Figure 3a). Despite not having EGFR mutations, this patient responded well to EGFR inhibitors afatinib, erlotinib, and gefitinib. Additionally, TR105 responded to nintedanib. Interestingly, this patient responded well to dabrafenib and trametinib, even though specific mutations indicating the use of these drugs were not identified through WES. The intrinsic expression of all three ABC transporters was lower than the threshold set at 20% (Figure 3b). 

TR36 (stage IIB, squamous-cell carcinoma, 65-year-old male with diabetes mellitus and chronic obstructive pulmonary disease, smoker) has a TP53 missense mutation c.701A>G characterized as likely pathogenic with moderate impact on protein function and reported in Li–Fraumeni syndrome and different cancer types including squamous-cell lung carcinoma; and a PIK3CA missense mutation c.1624G>A characterized as pathogenic with moderate impact on protein function and reported also in squamous-cell lung carcinoma. Besides erlotinib and nintedanib, this patient also responded well to dabrafenib (Figure 3a) although BRAF V600E mutation was not identified through WES. The intrinsic expression of all three ABC transporters was below 20% (Figure 3b). 

TR58 (stage IIA, adenocarcinoma, 70-year-old male with multiple sclerosis and diabetes mellitus, smoker) has a STAT5B frame-shift mutation c.1102del characterized as pathogenic with high impact on protein function and associated with immune dysregulation, including chronic pulmonary disease, interstitial pneumonitis, recurrent or severe infections, eczema, and autoimmune arthritis; and a BRCA1 splice-site mutation c.5075-1G>C characterized as pathogenic with high impact on disease development and reported in hereditary breast and ovarian cancer syndrome, and breast-ovarian familial cancer. This patient was sensitive only to erlotinib and nintedanib (Figure 3a), while the intrinsic expression of all three ABC transporters was higher than 20% (Figure 3b). TR58 has the second highest mutational burden in our cohort with the highest number of genes affected by multi-hit mutations (Figure 8).

TR93 (stage IIA, squamous-cell carcinoma, 72-year-old male with asthma, heavy smoker) has the same STAT5B frame-shift mutation c.1102del as TR58. TR93 responded to dabrafenib and erlotinib but not to nintedanib (Figure 3a). However, this patient has lower mutational burden than TR58 (Figure 8), while the intrinsic expression of all three ABC transporters was below 20% (Figure 3b).

TR100 (stage IIA, adenocarcinoma, 61-year-old male with occupation risk) has a TP53 non-sense mutation c.892G>T characterized as pathogenic with high impact on protein function and reported in rhabdomyosarcoma, neoplasm of ovary, hereditary cancer-predisposing syndrome, and Li–Fraumeni syndrome. This patient was sensitive to alectinib, dabrafenib, erlotinib, and nintedanib (Figure 3a). The intrinsic expression of ABCB1 and ABCC1 was less than 20%, while it was greater than 20% for ABCG2 (Figure 3b).

TR102 (stage IIB, adenocarcinoma, 55-year-old male with occupation risk, smoker) has a KRAS missense mutation c.35G>T characterized as pathogenic with moderate impact on protein function and reported in non-small cell lung carcinoma. This patient was sensitive only to erlotinib and nintedanib (Figure 3a). The intrinsic expression of ABCB1 and ABCC1 was less than 20%, while it was greater than 20% for ABCG2 (Figure 3b).

TR64 (stage IIIA, adenocarcinoma, 71-year-old male, non-smoker) has an EGFR missense mutation c.2573T>G characterized as pathogenic with moderate impact on protein function and reported in lung adenocarcinoma. This mutation is responsible for the changed efficacy of erlotinib and gefitinib. TR64 responded only to erlotinib and nintedanib, while the intrinsic expression of all three ABC transporters was greater than 20% (Figure 3b).

TR107 (stage IIIA, squamous-cell carcinoma, 77-year-old male with history of bladder carcinoma, former smoker) has a TP53 missense mutation c.743G>A characterized as pathogenic with moderated impact on protein function and reported in Li–Fraumeni syndrome, lung adenocarcinoma, and squamous-cell lung carcinoma. TR107 responded only to erlotinib and nintedanib (Figure 3a), while the intrinsic expression of ABCB1 was above 20% (Figure 3b).

TR109 (stage IVA, adenocarcinoma, 59-year-old male with meningeoma angiomatosum gradus 1) has a BAX frame-shift mutation c.121dup characterized as pathogenic and with high impact on protein function and reported in carcinoma of colon, and inborn genetic diseases. TR109 was sensitive only to erlotinib (Figure 3a), while the intrinsic expression of all three ABC transporters was lower than 20% (Figure 3b).

The Spearman’s correlation analysis of the results obtained, including sensitivity to TKIs (the number of TKIs to which the patient sample was sensitive), intrinsic resistance (the number of ABC transporters with high expression), the level of the mutational burden, and the presence of pathogenic prioritized mutations, revealed a significant negative correlation only between stage and sensitivity to TKIs. This suggests that patients with lower stages in our cohort responded to more TKIs (Figure S2, Tables S3 and S4). However, this result is not prominent, with Spearman r ≈ −0.6 indicating a relatively weak negative correlation. Additional patient samples are required to confirm this. Furthermore, sensitivity to TKIs and intrinsic resistance exhibited negative correlations with mutational burden, although these were not significant (Figure S2, Table S3). Additionally, pathogenic mutations showed a positive correlation with intrinsic resistance and mutational burden but without significance (Figure S2, Table S3).

## 4. Discussion

The influence of TKIs on the MDR phenotype is an important aspect of lung cancer therapy. Although TKIs effectively interfere with certain signaling pathways in cancer cells, they can also be substrates for ABC transporters [33,34]. At higher concentrations, TKIs can even act as inhibitors of these pumps. However, it is important to note that some TKIs, similar to conventional chemotherapeutic agents, can induce MDR by promoting the overexpression of ABC transporters [35,36]. This overexpression can become a significant problem in subsequent cancer treatment cycles. Therefore, our study aimed to correlate the functional testing of TKIs with the mutational profile of patients to better understand their impact on MDR and provide a basis for novel personalized therapeutic approaches.

NSCLC encompasses a diverse spectrum of genetic alterations, which has significant implications for therapeutic strategies. EGFR mutations, which occur in approximately 32% of NSCLC cases worldwide, are particularly common in the adenocarcinoma subtype [37]. At the same time, KRAS mutations are observed in up to 30% of NSCLC cases and represent another important driving alteration [38]. TP53 mutations are detected in approximately 50% of NSCLC cases, highlighting their substantial presence and potential impact on disease progression and response to treatment [39]. In our sample group, we identified only one patient with an EGFR mutation and one with a KRAS mutation (both patients have adenocarcinoma). However, 11 patients carried TP53 mutations, which accounts for 65% patients in our NSCLC cohort. The evaluation of the sensitivity of 10 TKIs in patient-derived NSCLC cultures revealed different responses. The TKIs were applied at clinically relevant concentrations to mimic real-life treatment scenarios. Erlotinib emerged as the most promising TKI due to its consistent and substantial efficacy in the investigated patient samples. Erlotinib decreased the number of cancer cells (CK8/18+ cells) in all NSCLC cultures. In addition, erlotinib exclusively inhibited the growth of cancer cells in NSCLC cultures while sparing stromal cells (CK8/18− cells). Seemingly, this selective effect qualifies erlotinib as a valuable therapeutic option but after its treatment, the expression of ABC transporters increased in several samples. In almost all patient-derived NSCLC cells, erlotinib showed an anticancer effect at lower concentrations without a dose–response effect, indicating that higher concentrations will not provide additional benefit. This discovery has the potential to lead to individualized treatment recommendations for erlotinib.

We also assayed the impact of stromal cells, mainly fibroblasts, on the TKI sensitivity of NSCLC patient samples. Our observations revealed diverse responses of stromal cells to TKIs among different patients. Notably, erlotinib demonstrated a significant effect, showing the complete non-responsiveness of stromal cells in most patients and selectivity towards cancer cells in all patients. The reduction in stromal cells could be only observed in TR64 with an EGFR mutation and TR58 with a high mutational burden. The resistance to other EGFR inhibitors (afatinib, gefitinib, and osimertinib) in our patient cohort may be linked to the continuous exposure of primary cell cultures to exogenous EGF in the culturing medium. Erlotinib was able to surpass this limitation. Additionally, EGF secretion by cancer-associated fibroblasts could contribute to resistance in neighboring cancer cells, a challenge that erlotinib, unlike other EGFR inhibitors tested, could overcome.

Erlotinib is generally used to treat NSCLC with constitutively activated EGFR due to point mutations [40,41,42,43,44,45]. A significant proportion of NSCLCs with EGFR mutations responded positively to EGFR tyrosine kinase inhibitors such as erlotinib, while a significantly lower response was observed in tumors without EGFR mutations [41]. In contrast, our results deviate from these conclusions. A number of cultures derived from patients with wild-type EGFR responded better to erlotinib than culture TR64 with an EGFR L858R mutation [22]. These results demonstrate the broad efficacy of erlotinib in all primary cultures and emphasize its potential beyond the conventional EGFR-mutated subgroup. Our results are also consistent with the findings of other studies [46,47,48,49,50,51,52] demonstrating the efficacy of erlotinib in a diverse NSCLC patient population regardless of EGFR status, NSCLC type (adenocarcinoma or squamous-cell carcinoma), TMB, and chemotherapy history.

We found that erlotinib significantly increased ABCG2 expression in a subset of CK8/18+ cultures, namely, TR28, TR64, TR102, TR105, and TR107, suggesting that drug resistance may be induced after erlotinib application. Despite its widespread use as an EGFR tyrosine kinase inhibitor, erlotinib’s ability to modulate MDR mechanisms may pose a challenge in the treatment of NSCLC patients. Previous studies suggest that erlotinib does not increase ABCG2 expression [53,54]. On the contrary, there is evidence that erlotinib can act as a potent inhibitor of ABCB1 and ABCG2 function [55]. In contrast to erlotinib, most other TKIs showed no effect on cancer cells in NSCLC cultures. The effect of the TKIs on non-cancer cells varied from culture to culture, with dabrafenib being effective in both CK8/18+ and CK8/18− cells, crizotinib primarily targeting stromal cells, and nintedanib showing a strong response in stromal cell populations.

Nintedanib, an oral tyrosine kinase inhibitor targeting VEGFR1-3, FGFR1-3, and PDGFR, is approved for the treatment of idiopathic pulmonary fibrosis and lung cancer [56,57,58]. Nintedanib showed an unexpected effect on cancer cells, stimulating their growth in some of NSCLC cultures at lower concentrations, while inhibiting it at higher concentrations. The stromal cell population, which consists mainly of fibroblasts, was more sensitive to nintedanib at lower concentrations than the cancer cells. This is in line with expectations, considering that nintedanib is used to treat idiopathic pulmonary fibrosis, in which pathogenesis fibroblasts play a central role.

Nintedanib enriched the population of CK8/18+ cells in ABCB1-, ABCC1-, and ABCG2-positive cells in most examined NSCLC cultures. Nintedanib increased ABCG2-positive CK8/18+ cells at concentrations that were not particularly cytotoxic in TR80, TR93, and TR107 cultures. In addition, ABCB1-positive CK8/18+ cells increased in TR93 and TR100, while ABCC1-positive CK8/18+ cells increased in TR80 and TR93, at nintedanib concentrations without significant cytotoxic effects. Cancer cells with a high expression of MDR markers that survive treatment with nintedanib raise concerns about nintedanib application, particularly in combined treatments with other anticancer drugs—ABC transporters’ substrates.

Aiming at understanding the relationship between the expression of ABC transporters and the sensitivity to different TKIs, we categorized our samples into two groups—sensitive and resistant—based on the presence of ABC transporter-positive cells before TKI treatment. However, when the responses of the NSCLC cells to the TKIs were compared to the inherent expression of ABC transporters in control cells, only 8 cultures out of 17 fitted into these defined categories. The findings indicate that the sensitivity of TKIs in our NSCLC cultures derived from patients is not attributed to the inherent expression of ABC transporters. Combining a TKI with an inhibitor that targets ABCB1, ABCC1, or ABCG2 can be a potentially effective strategy to gain insights into the role of MDR mechanism, which is mediated by ABC transporters, in determining TKI sensitivity.

Most stromal cells in NSCLC cultures showed no intrinsic expression of ABCB1, ABCC1, or ABCG2 and showed only a slight increase in expression of these MDR markers after treatment with erlotinib or nintedanib, which was not significant, indicating that stromal cells do not take part in the development of MDR to tested TKIs.

Studies linking stromal cells with a poor response to chemotherapy only imply an association rather than establishing a direct cause [59]. Stromal cells may promote the outgrowth of cancer-resistant clones and increase AKT pro-survival signaling through soluble growth factor secretion. Efforts to establish these mechanistic connections are ongoing and are of great interest in improving the effectiveness of anticancer therapies. Co-targeting stromal and cancer cells could potentially harness stroma-induced synthetic lethality pathways. Our findings did not reveal associations between cancer and stromal cells treated simultaneously with TKIs. Notably, stromal cells in most patient samples had a neglectable intrinsic expression of ABC transporters, which was not significantly stimulated by TKIs.

In our study, the limited effect of most TKIs on the expression of MDR markers in cancer and normal cells was consistent with their observed low efficacy in NSCLC cultures. Although afatinib, alectinib, ceritinib, crizotinib, dabrafenib, gefitinib, osimertinib, and trametinib are substrates for ABC transporters [33,34], they may not significantly induce their expression. The increase in ABCG2 expression in NSCLC culture TR107 following treatment with alectinib, ceritinib, dabrafenib, erlotinib, and nintedanib highlights the unique response of this specific culture and illustrates the patient-dependent nature of drug-induced changes in ABC transporter expression. Despite the lack of response to alectinib, ceritinib, and dabrafenib in TR107, these TKIs increased the ABCG2 expression after treatment.

The observed increase in ABC transporter expression after 7 days of TKI treatment raises concerns that the clinical efficacy of TKIs may decline rapidly. While our assay suggests the possibility of an increase in MDR, it does not clarify whether this increase is transient or permanent, nor does it provide information on the magnitude of this increase. Instead, it indicates a tendency for certain patients to develop resistance to certain TKIs, suggesting that combining these TKIs with other therapies may not benefit these patients. Various mechanisms such as epigenetics [60] and extracellular vesicles carrying MDR-inducing components [61,62,63] could contribute to the rapid emergence of a resistant phenotype in NSCLC cultures.

Five NSCLC cultures showed a positive response to dabrafenib, although none of these cultures had the BRAF V600E mutation. Among these, only TR87 showed an intrinsic expression of ABC transporters and sensitivity to dabrafenib. In TR105, TR36, TR93, and TR100, however, less than 20% of the cells were positive for ABC transporters. Remarkably, TR105 responded to both dabrafenib and trametinib, making it a potential candidate for combination therapy despite the absence of a BRAF mutation. Interestingly, two out of three cultures from stage I patients responded well to afatinib. In addition, the response to erlotinib remained good regardless of the intrinsic expression of the ABC transporters in the control groups. Mutations in TP53 can have far-reaching effects as it is involved in several signaling pathways that play a role in NSCLC development and response to treatment [64,65,66].

Wild-type p53 is known to suppress ABCB1 gene transcription by binding directly to the ABCB1 gene promoter, whereas mutant p53 enhances ABCB1 promoter activity [67]. However, it was not possible to establish a clear-cut correlation between TP53 mutations and resistance to TKIs because a significant number of patients with TP53 mutations (8 out of 11) responded positively to two TKIs, while a substantial number of patients with wtTP53 (4 out of 6) also responded to two TKIs.

Nine NSCLC patients possess clinically described and significant mutations in prioritized genes involved in enriched KEGG pathways (TP53, PIK3CA, STATB5, BRCA1, KRAS, EGFR, and BAX). The same deletion in STAT5B was identified in TR58 and TR93 samples, which responded only to two TKIs. TR102 with a KRAS mutation and TR64 with an EGFR mutation also responded to only two TKIs. TR109, the only stage IV NSCLC in our cohort, has mutated BAX with a frame-shift insertion, and this patient only responded to erlotinib.

It is important to note that we lack performance data on the patients as the most of them did not receive targeted therapy. TR28, who received two cycles of the combination of gemcitabine and cisplatin as neoadjuvant chemotherapy, has the most prominent initial expression of all three ABCB1 transporters, particularly ABCC1 (in both cancer and stromal cells) that is responsible for cisplatin-induced drug resistance [68].

Only TR64 received osimertinib as adjuvant therapy. While our study provides valuable insight into the effects of TKIs on patients’ NSCLC cultures, it is essential to consider the limited clinical context when applying these findings to clinical situations. According to the current guidelines, the use of osimertinib is a preferred treatment option for patients with completely resected stage IB–IIIA or IIIB (T3 and N2) NSCLC and positive for EGFR mutations (exon 19 deletion and exon 21 L858R) who have previously received adjuvant chemotherapy or are not eligible for platinum-based chemotherapy [69,70]. This form of treatment is currently not supported by the Republic Fund of Health Insurance in Serbia, and TKIs are administered in later stages (IIIb and IV). The NSCLC cultures we tested did not respond to osimertinib, including TR64 with the EGFR L858R mutation, which is in contrast to erlotinib, where all cultures showed sensitivity. There is the potential for personalized treatment selection based on individual patient responses. Our results encourage further exploration of the potential benefits and challenges associated with the use of TKIs in the treatment of early-stage NSCLC in Serbia. They highlight the need for personalized treatment strategies based on individual patient profiles.

## 5. Conclusions

Our research indicates that in addition to mutational status, it is necessary to perform functional sensitivity screening to determine the appropriate treatment approaches with TKIs. We analyzed the causalities between ABC transporter expression, gene alterations, and sensitivity to a particular TKI for each patient. Nevertheless, we could not identify common response patterns. Interestingly, erlotinib was equally effective in patient samples with low and high TMB regardless of EGFR status, challenging the conventional view that erlotinib is most effective in EGFR-mutated NSCLC. Furthermore, we found more options among TKIs for treatment of NSCLC lower stages. Out of the 17 NSCLC patients analyzed, 9 exhibited gene mutations that are clinically relevant. This highlights the concern that many NSCLC patients may not have well-defined biomarkers to predict their response to TKIs. The ultimate factor that matters is the sensitivity of NSCLC patients to specific TKIs. Our ex vivo testing, combined with MDR profiling, provides a reliable foundation for the personalized therapy of NSCLC patients who do not have clinically relevant biomarkers. This approach can potentially benefit a significant portion of NSCLC patients who do not have gene mutations that can be treated by targeted therapeutics, and can thus pave the way for the successful personalized treatment of NSCLC patients.

## Figures and Tables

**Table 1:** IC_50_ values of tyrosine kinase inhibitors in NSCLC patient-derived cultures.

NSCLC Cultures		IC_50_ (µM)									
Cancer Cells (CK8/18+)		Afatinib	Alectinib	Ceritinib	Crizotinib	Dabrafenib	Erlotinib	Gefitinib	Nintedanib	Osimertinib	Trametinib
Stage I	TR84	0.0447	>1.5	>1.5	>1	>5	2.430	>0.4 [22]	12.851	>0.15	>0.025
	TR104	>0.05	>1.5	>1.5	>1	>5	3.250	>0.4	17.525	>0.15	>0.025
	TR105	0.0293	>1.5	>1.5	>1	2.595	0.456	0.399	3.240	>0.15	0.024
Stage II	TR28	>0.05	N/A *	>1.5	>1	>5	0.240	>0.4	8.685	>0.15	>0.025
	TR36	>0.05	>1.5	>1.5	>1	4.381	2.288	>0.4	19.315	>0.15	>0.025
	TR58	>0.05	>1.5	>1.5	>1	>5	1.829	>0.4 [22]	13.631	>0.15	>0.025
	TR80	>0.05	>1.5	>1.5	>1	>5	3.276	>0.4	18.125	>0.15	>0.025
	TR87	>0.05	>1.5	>1.5	>1	0.608	1.297	>0.4 [22]	15.869	>0.15	>0.025
	TR93	>0.05	>1.5	>1.5	>1	>5	3.291	>0.4	>20	>0.15	>0.025
	TR100	>0.05	1.391	>1.5	>1	4.299	2.630	>0.4	4.939	>0.15	>0.025
	TR102	>0.05	>1.5	>1.5	>1	>5	0.916	>0.4	17.862	>0.15	>0.025
	TR106	>0.05	>1.5	>1.5	>1	>5	2.123	>0.4	20.174	>0.15	>0.025
Stage III	TR33	>0.05	>1.5	>1.5	>1	>5	0.969	>0.4	16.706	>0.15	>0.025
	TR34	>0.05	>1.5	>1.5	>1	>5	1.385	>0.4	10.901	>0.15	>0.025
	TR64	>0.05	>1.5	>1.5	>1	>5	2.765	>0.4 [22]	10.596	>0.15	>0.025
	TR107	>0.05	>1.5	>1.5	>1	>5	2.070	>0.4	17.625	>0.15	>0.025
Stage IV	TR109	>0.05	>1.5	>1.5	>1	>5	3.028	>0.4	>20	>0.15	>0.025
Non-Cancer Cells (CK8/18−)		Afatinib	Alectinib	Ceritinib	Crizotinib	Dabrafenib	Erlotinib	Gefitinib	Nintedanib	Osimertinib	Trametinib
Stage I	TR84	>0.05 ^s^	>1.5	>1.5	>1	>5	>4 ^s^	>0.4 [22]	4.161	>0.15	>0.025
	TR104	>0.05	>1.5	>1.5	>1	>5	>4 ^s^	>0.4	7.481	>0.15	>0.025
	TR105	>0.05 ^s^	>1.5	>1.5	>1	>5 ^s^	>4 ^s^	>0.4 ^s^	0.618	>0.15	>0.025 ^s^
Stage II	TR28	>0.05	N/A *	>1.5	>1	>5	>4 ^s^	>0.4	10.624	>0.15	>0.025
	TR36	>0.05	>1.5	>1.5	0.726	1.044	>4 ^s^	>0.4	0.909	>0.15	>0.025
	TR58	>0.05	>1.5	>1.5	>1	>5	3.205 ^s^	>0.4 [22]	5.215	>0.15	>0.025
	TR80	>0.05	1.447	>1.5	0.404	4.531	>4 ^s^	>0.4	2.732	0.133	>0.025
	TR87	>0.05	>1.5	>1.5	0.927	0.229	>4 ^s^	0.188 [22]	1.069	>0.15	>0.025
	TR93	>0.05	>1.5	>1.5	>1	>5	>4 ^s^	>0.4	>20	>0.15	0.005
	TR100	>0.05	0.403	0.358	0.176	0.787	>4 ^s^	>0.4	0.357	>0.15	>0.025
	TR102	>0.05	>1.5	>1.5	>1	>5	>4 ^s^	>0.4	6.559	>0.15	>0.025
	TR106	>0.05	>1.5	>1.5	>1	>5	>4 ^s^	>0.4	2.112	>0.15	>0.025
Stage III	TR33	>0.05	>1.5	>1.5	>1	>5	>4 ^s^	>0.4	6.682	>0.15	>0.025
	TR34	>0.05	>1.5	>1.5	0.921	>5	>4 ^s^	>0.4	1.568	>0.15	>0.025
	TR64	>0.05	>1.5	>1.5	>1	>5	>4 ^s^	>0.4 [22]	7.360	>0.15	>0.025
	TR107	>0.05	>1.5	>1.5	>1	>5	>4 ^s^	>0.4	4.150	>0.15	>0.025
Stage IV	TR109	>0.05	>1.5	>1.5	>1	>5	>4 ^s^	>0.4	6.010	>0.15	>0.025

^s^ Selectivity towards cancer cells. * TR28 alectinib treatment data were excluded due to poor quality.

**Table 2:** NSCLC patient-derived cultures with increased ABCB1, ABCC1, and ABCG2 expression after treatment with tyrosine kinase inhibitors.

CK8/18+ Cells	ABCB1 * Expression Increased	ABCC1 * Expression Increased	ABCG2 * Expression Increased
TR28	erlotinib, nintedanib	erlotinib	erlotinib, nintedanib
TR33	/	/	/
TR34	/	/	nintedanib
TR36	/	/	nintedanib
TR58	nintedanib	nintedanib	/
TR64	nintedanib	erlotinib, nintedanib	erlotinib, nintedanib
TR80	/	dabrafenib, nintedanib	nintedanib
TR84	gefitinib, nintedanib	gefitinib [22], nintedanib	gefitinib, nintedanib
TR87	ceritinib, erlotinib, nintedanib	afatinib, nintedanib	ceritinib, gefitinib, nintedanib
TR93	nintedanib	nintedanib	nintedanib
TR100	nintedanib	nintedanib	nintedanib
TR102	/	/	erlotinib
TR104	/	/	/
TR105	nintedanib	nintedanib	erlotinib, nintedanib
TR106	/	/	nintedanib
TR107	/	/	alectinib, ceritinib, dabrafenib, erlotinib, nintedanib
TR109	/	/	/

* The table shows an increase in the expression of ABCB1, ABCC1, and ABCG2 by at least 20% at one or more concentrations.

## Data Availability

The data presented in this study are available upon reasonable request to the corresponding author.

## Supplementary Materials

The following supporting information can be downloaded at: https://www.mdpi.com/article/10.3390/cancers16111984/s1, Table S1. Clinical parameters of patients with NSCLC; Table S2. Mutated genes in NSCLC patients assessed through WES and their involvement in some of the signaling pathways identified using KEGG analysis; Table S3. Spearman rank analysis illustrating positive or negative correlations between different variables in 17 samples from NSCLC patients. Table S4. Ranking of different variables in 17 samples from NSCLC patients. Figure S1. Mutation summary in NSCLC patients. Figure S2. Spearman’s correlation analysis. Correlations and statistical significance were examined among different variables in 17 NSCLC patient samples, including stage, sensitivity to TKIs, intrinsic resistance, mutational burden, and pathogenic mutations.
