# Supplementary material for: Multidrug-Resistant Profiles in Non-Small Cell Lung Carcinoma Patient-Derived Cells: Implications for Personalized Approaches with Tyrosine Kinase Inhibitors
Source: Cancers (Basel). 2024 May 23;16(11):1984. doi: 10.3390/cancers16111984 (PMC11171162; doi:10.3390/cancers16111984)
Supplement: Supplementary file 1 [file cancers-16-01984-s001.zip › cancers-3008579-supplementary.pdf]

Table S1. Clinical parameters of patients with NSCLC.

Patient	Sex	Age	NSCLC subtype	Lymph node invasion	Stage	EGFR status	PFS	OS	NAC
TR28	male	67	squamous cell carcinoma	N0	IIB	wt	> 12 months	/	gemcitabine + cisplatin, 2 cycles
TR33	male	47	squamous cell carcinoma	N0	IIIA	wt	> 12 months	/	no
TR34	male	73	squamous cell carcinoma	N2	IIIB	wt	2.2 months	2.8 months	no
TR36	male	65	squamous cell carcinoma	N0	IIB	wt	> 12 months	/	no
TR58	female	70	adenocarcinoma	N0	IIA	wt	> 12 months	/	no
TR64	male	71	adenocarcinoma	N2	IIIA	L858R	> 12 months	/	no
TR80	female	79	squamous cell carcinoma	N0	IIB	wt	> 12 months	/	no
TR84	female	58	squamous cell carcinoma	N0	IB	wt	N/A	/	no
TR87	male	68	squamous cell carcinoma	N0	IIB	wt	N/A	/	no
TR93	male	72	squamous cell carcinoma	N0	IIA	wt	N/A	/	no
TR100	male	61	adenocarcinoma	N0	IIA	wt	N/A	/	no
TR102	male	55	adenocarcinoma	N0	IIB	wt	N/A	/	no
TR104	male	68	adenocarcinoma	N0	IB	wt	N/A	/	no
TR105	male	66	squamous cell carcinoma	N0	IB	wt	N/A	/	no
TR106	female	74	squamous cell carcinoma	N0	IIB	wt	N/A	/	no
TR107	male	77	squamous cell carcinoma	N2	IIIA	wt	N/A	/	no
TR109	male	59	adenocarcinoma	N1	IVA	wt	N/A	/	no

N – node
PFS – progression free survival
OS – overall survival
NAC – neoadjuvant chemotherapy
N/A – surgery less than 12 months ago

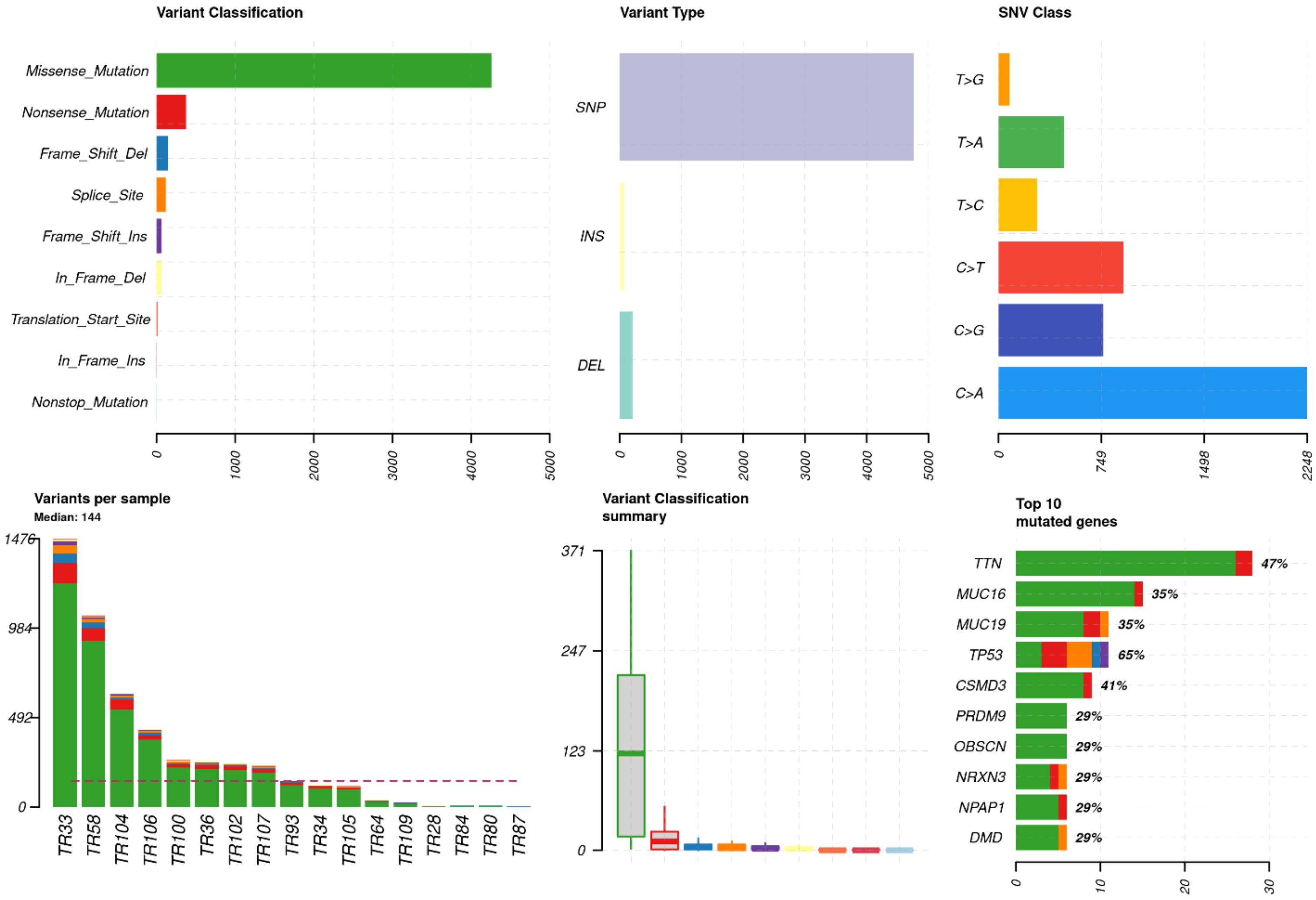

Figure S1. Mutation summary in NSCLC patients. The maftools plot shows a summary of the MAF file. The most frequently mutated genes, the SNV class and the distribution of variant classification in NSCLC patients are highlighted.

Table S2. Mutated genes in NSCLC patients assessed by WES and their involvement in some of the signaling pathways identified by KEGG analysis.

**Patients***		Proteoglycans in cancer	PI3K-Akt signaling pathway	Transcriptional misregulation in cancer	Rap1 signaling pathway	Ras signaling pathway	Calcium signaling pathway	EGFR tyrosine kinase inhibitor resistance	Focal adhesion	Chemical carcinogenesis - receptor activation	Central carbon metabolism in cancer	ErbB signaling pathway	Platinum drug resistance	Signaling pathways regulating pluripotency of stem cells	HIF-1 signaling pathway
Stage I	TR104	**ERBB4**<br>c.1715C>T,<br>**PRKACA**<br>c.1039G>C<br>**TP53**<br>c.919+1G>T	**ERBB4**<br>c.1715C>T<br>**TP53**<br>c.919+1G>T	**BCL6**<br>c.2052C>G<br>**EWSR1**<br>c.1889G>A<br>**TP53**<br>c.919+1G>T<br>**ZEB1**<br>c.1264G>C	–	**PRKACA**<br>c.1039G>C	**ERBB4**<br>c.1715C>T<br>**PRKACA**<br>c.1039G>C	–	**ARHGAP35**<br>c.3238G>A	**BCL6**<br>c.2052C>G<br>**PRKACA**<br>c.1039G>C	**TP53**<br>c.919+1G>T	**ERBB4**<br>c.1715C>T	**MSH6**<br>c.188C>G<br>**TP53**<br>c.919+1G>T	–	–
	TR105	**TP53**<br>c.637C>T	**CSF3R**<br>c.2034G>A<br>**TP53**<br>c.637C>T	**SS18**<br>c.1127A>T<br>**TP53**<br>c.637C>T	–	–	–	–	–	–	**TP53**<br>c.637C>T	–	**TP53**<br>c.637C>T	–	–
Stage II	TR36	**CDKN1A**<br>c.137G>C<br>**KDR**<br>c.2029A>T<br>**PIK3CA**<br>c.1624G>A<br>**TP53**<br>c.701A>G	**CDKN1A**<br>c.137G>C<br>**KDR**<br>c.2029A>T<br>**PIK3CA**<br>c.1624G>A<br>**TP53**<br>c.701A>G	**CDKN1A**<br>c.137G>C<br>**NR4A3**<br>c.1576A>T<br>**TP53**<br>c.701A>G	**KDR**<br>c.2029A>T<br>**PIK3CA**<br>c.1624G>A	**KDR**<br>c.2029A>T<br>**PIK3CA**<br>c.1624G>A	**KDR**<br>c.2029A>T	**KDR**<br>c.2029A>T<br>**PIK3CA**<br>c.1624G>A	**KDR**<br>c.2029A>T<br>**PIK3CA**<br>c.1624G>A	**KLF4**<br>c.1354G>T<br>**PIK3CA**<br>c.1624G>A	**PIK3CA**<br>c.1624G>A<br>**TP53**<br>c.701A>G	**CDKN1A**<br>c.137G>C<br>**PIK3CA**<br>c.1624G>A	**CDKN1A**<br>c.137G>C<br>**PIK3CA**<br>c.1624G>A<br>**TP53**<br>c.701A>G	**KLF4**<br>c.1354G>T<br>**PIK3CA**<br>c.1624G>A	**CDKN1A**<br>c.137G>C<br>**PIK3CA**<br>c.1624G>A
	TR58	**HGF**<br>c.944G>T<br>**MET**<br>c.1545G>T<br>**SMO**<br>c.1579G>A<br>**VAV1**<br>c.2039G>T	**BRCA1**<br>c.5075-1G>C<br>**HGF**<br>c.944G>T<br>**MET**<br>c.1545G>T<br>**NTRK2**<br>c.1339C>A<br>**TCL1A**<br>c.277C>A	**MET**<br>c.1545G>T<br>**PBX1**<br>c.373G>T	**GNAS**<br>c.1757C>A<br>**HGF**<br>c.944G>T<br>**MET**<br>c.1545G>T<br>**VAV1**<br>c.2039G>T	**HGF**<br>c.944G>T<br>**MET**<br>c.1545G>T<br>**NTRK2**<br>c.1339C>A	**GNAS**<br>c.1757C>A<br>**HGF**<br>c.944G>T<br>**MET**<br>c.1545G>T<br>**NTRK2**<br>c.1339C>A	**HGF**<br>c.944G>T<br>**MET**<br>c.1545G>T	**HGF**<br>c.944G>T<br>**MET**<br>c.1545G>T<br>**VAV1**<br>c.2039G>T	**GNAS**<br>c.1757C>A<br>**STAT5B**<br>c.1102del	**MET**<br>c.1545G>T	**STAT5B**<br>c.1102del	**BRCA1**<br>c.5075-1G>C	**TBX3**<br>c.1775C>A	–
	TR93	**TP53**<br>c.376-2A>T	**TP53**<br>c.376-2A>T	**TP53**<br>c.376-2A>T	–	–	–	–	–	**STAT5B**<br>c.1102del	**IDH1**<br>c.341G>T<br>**TP53**<br>c.376-2A>T	**STAT5B**<br>c.1102del	**TP53**<br>c.376-2A>T	–	–
	TR100	**TP53**<br>c.892G>T	**TP53**<br>c.892G>T	**TP53**<br>c.892G>T	–	–	–	–	–	–	**TP53**<br>c.892G>T	–	**TP53**<br>c.892G>T	–	–

	TR102	**KRAS**<br>c.35G>T<br>**SDC4**<br>c.13C>T<br>**TP53**<br>c.707A>G	**KRAS**<br>c.35G>T<br>**TP53**<br>c.707A>G	**BCL11B**<br>c.311C>G<br>**TP53**<br>c.707A>G	**KRAS**<br>c.35G>T	**KRAS**<br>c.35G>T	–	**KRAS**<br>c.35G>T	–	**KRAS**<br>c.35G>T	**KRAS**<br>c.35G>T<br>**TP53**<br>c.707A>G	**KRAS**<br>c.35G>T	**TP53**<br>c.707A>G	**KRAS**<br>c.35G>T	–
	TR106	**COL1A1**<br>c.3751A>G<br>**HIF1A**<br>c.203G>T<br>**PIK3R1**<br>c.418G>A	**COL1A1**<br>c.3751A>G<br>**PIK3R1**<br>c.418G>A<br>**PTEN**<br>c.402G>A	**ETV5**<br>c.340G>C	**PIK3R1**<br>c.418G>A	**PIK3R1**<br>c.418G>A	–	**PIK3R1**<br>c.418G>A<br>**PTEN**<br>c.402G>A	**COL1A1**<br>c.3751A>G<br>**PIK3R1**<br>c.418G>A<br>**PTEN**<br>c.402G>A	**PIK3R1**<br>c.418G>A	**HIF1A**<br>c.203G>T<br>**PIK3R1**<br>c.418G>A<br>**PTEN**<br>c.402G>A	**PIK3R1**<br>c.418G>A	**PIK3R1**<br>c.418G>A	**PIK3R1**<br>c.418G>A	**HIF1A**<br>c.203G>T<br>**TFRC**<br>c.1273C>G
Stage III	TR33	**ANK1**<br>c.2979C>G<br>**CTNNB1**<br>c.674G>T<br>**ERBB4**<br>c.1847C>A<br>**ESR1**<br>c.104T>A<br>**HGF**<br>c.2551G>T<br>**PRKCB**<br>c.1045G>T<br>**SMAD2**<br>c.548A>T	**CASP9**<br>c.1123G>T<br>**CREB3L1**<br>c.1352G>T<br>**ERBB4**<br>c.1847C>A<br>**HGF**<br>c.2551G>T	**ATM**<br>c.5443G>T<br>**PAX7**<br>c.223C>A,<br>c.1223C>A<br>**TLX3**<br>c.80C>A	**CTNNB1**<br>c.674G>T<br>**GRIN2A**<br>c.1045G>T<br>**HGF**<br>c.2551G>T<br>**PRKCB**<br>c.1045G>T	**GRIN2A**<br>c.1045G>T<br>**HGF**<br>c.2551G>T<br>**PRKCB**<br>c.1045G>T	**ERBB4**<br>c.1847C>A<br>**GRIN2A**<br>c.1045G>T<br>**HGF**<br>c.2551G>T<br>**NFATC2**<br>c.1901A>T<br>**PRKCB**<br>c.1045G>T	**HGF**<br>c.2551G>T<br>**PRKCB**<br>c.1045G>T	**CTNNB1**<br>c.674G>T<br>**HGF**<br>c.2551G>T<br>**PRKCB**<br>c.1045G>T	**CREB3L1**<br>c.1352G>T<br>**ESR1**<br>c.104T>A<br>**PRKCB**<br>c.1045G>T	–	**ERBB4**<br>c.1847C>A<br>**PRKCB**<br>c.1045G>T	**ATM**<br>c.5443G>T<br>**CASP9**<br>c.1123G>T	**CTNNB1**<br>c.674G>T<br>**TBX3**<br>c.1775C>A	**PRKCB**<br>c.1045G>T
	TR34	**PIK3R1**<br>c.1676T>G<br>**TP53**<br>c.184G>T	**PIK3R1**<br>c.1676T>G<br>**TP53**<br>c.184G>T	**TP53**<br>c.184G>T	**PIK3R1**<br>c.1676T>G	**PIK3R1**<br>c.1676T>G	–	**PIK3R1**<br>c.1676T>G	**PIK3R1**<br>c.1676T>G	**PIK3R1**<br>c.1676T>G	**PIK3R1**<br>c.1676T>G<br>**TP53**<br>c.184G>T	**PIK3R1**<br>c.1676T>G	**PIK3R1**<br>c.1676T>G<br>**TP53**<br>c.184G>T	**PIK3R1**<br>c.1676T>G	**PIK3R1**<br>c.1676T>G
	TR64	**EGFR**<br>c.2573T>G	**EGFR**<br>c.2573T>G	–	**EGFR**<br>c.2573T>G	**EGFR**<br>c.2573T>G	**EGFR**<br>c.2573T>G	**EGFR**<br>c.2573T>G	**EGFR**<br>c.2573T>G	**EGFR**<br>c.2573T>G	**EGFR**<br>c.2573T>G	**EGFR**<br>c.2573T>G	–	–	**EGFR**<br>c.2573T>G
	TR107	**PLCG1**<br>c.3487G>C<br>**TP53**<br>c.743G>A	**FGFR3**<br>c.1477G>T<br>**JAK3**<br>c.2495A>G<br>**TP53**<br>c.743G>A	**TGFBR2**<br>c.251G>A<br>**TP53**<br>c.743G>A<br>**ZEB1**<br>c.806A>G	**FGFR3**<br>c.1477G>T<br>**PLCG1**<br>c.3487G>C	**ABL1**<br>c.1893A>T<br>**FGFR3**<br>c.1477G>T<br>**PLCG1**<br>c.3487G>C	**FGFR3**<br>c.1477G>T<br>**PLCG1**<br>c.3487G>C	**FGFR3**<br>c.1477G>T<br>**PLCG1**<br>c.3487G>C	–	–	**FGFR3**<br>c.1477G>T<br>**TP53**<br>c.743G>A	**ABL1**<br>c.1893A>T<br>**PLCG1**<br>c.3487G>C	**TP53**<br>c.743G>A	**FGFR3**<br>c.1477G>T	**PLCG1**<br>c.3487G>C
Stage IV	TR109	–	–	**BAX**<br>c.121dup	–	–	–	**BAX**<br>c.121dup	–	–	–	–	**BAX**<br>c.121dup	–	–

* Samples TR84 (stage I), TR28, TR80 and TR87 (stage II) were not identified by variant prioritization and pathway analysis

Highlighted gene mutations have clinical significance according to ClinVar database.

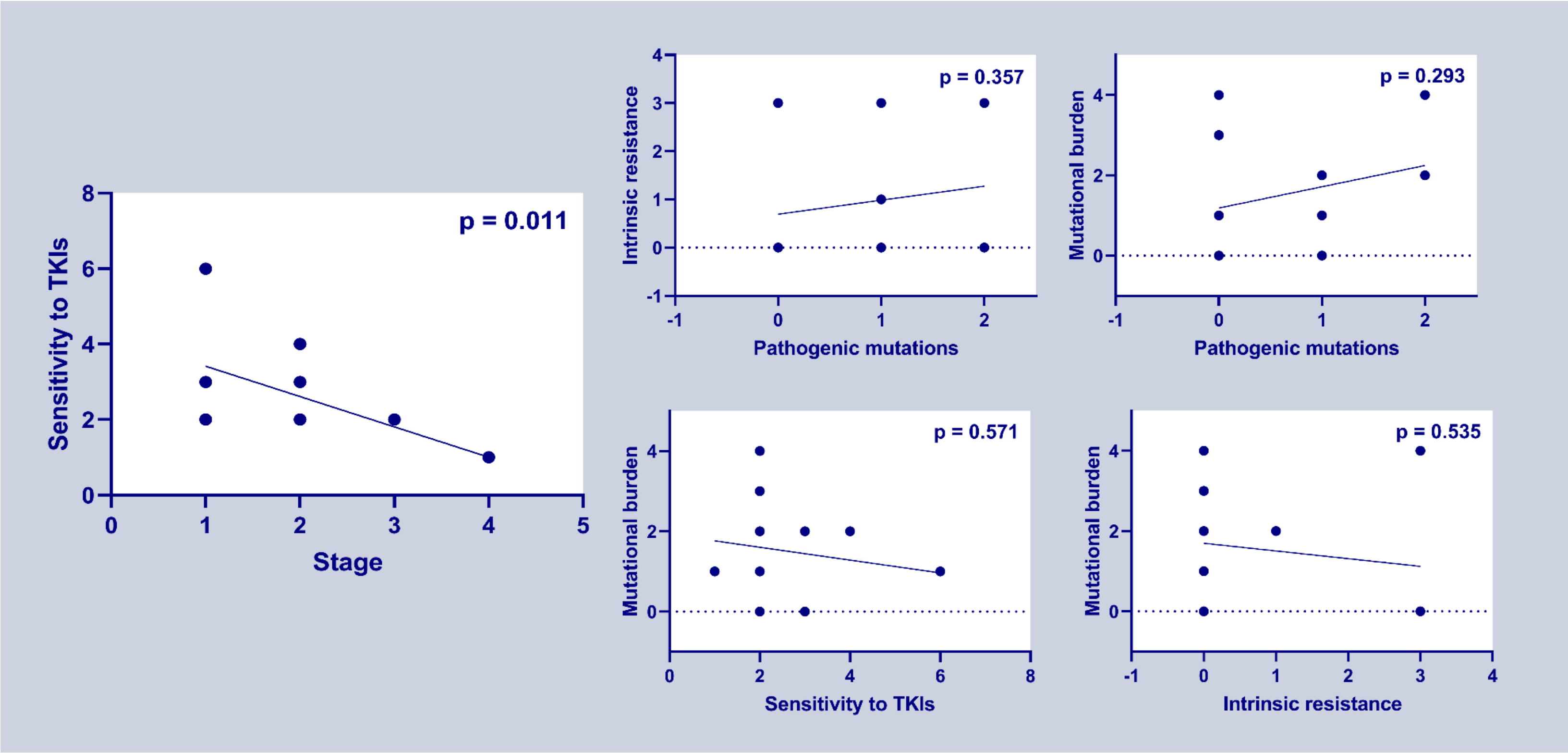

Figure S2. Spearman's correlation analysis. Correlations and statistical significance were examined among different variables in 17 NSCLC patient samples, including stage, sensitivity to TKIs, intrinsic resistance, mutational burden, and pathogenic mutations.

Table S3. Spearman rank analysis illustrating positive or negative correlations between different variables in 17 samples from NSCLC patients.

Spearman r	*Stage*	*Sensitivity to TKIs*	*Intrinsic resistance*	*Mutational burden*	*Pathogenic mutations*
Stage	1	-0.598*	0.145	0.041	0.128
Sensitivity to TKIs	-0.598*	1	0.031	-0.148	0.090
Intrinsic resistance	0.145	0.031	1	-0.160	0.224
Mutational burden	0.041	-0.148	-0.160	1	0.270
Pathogenic mutations	0.128	0.090	0.224	0.270	1

*$p < 0.05$

Table S4. Ranking of different variables in 17 samples from NSCLC patients.

	Stage [a]	*Sensitivity to TKIs* [b]	*Intrinsic resistance* [c]	*Mutational burden* [d]	*Pathogenic mutations* [e]
TR84	1	3	0	0	0
TR104	1	2	0	3	0
TR105	1	6	0	1	1
TR28	2	2	3	0	0
TR36	2	3	0	2	2
TR58	2	2	3	4	2
TR80	2	2	0	0	0
TR87	2	3	3	0	0
TR93	2	2	0	1	1
TR100	2	4	1	2	1
TR102	2	2	1	2	1
TR106	2	2	0	3	0
TR33	3	2	0	4	0
TR34	3	2	0	1	0
TR64	3	2	3	0	1
TR107	3	2	1	2	1
TR109	4	1	0	1	1

[a] Stages ranging from 1 to 4

[b] Sensitivity to TKIs [b] from 1 to 6 (number of TKIs to which patient sample was sensitive)

[c] Intrinsic resistance from 0 to 3 (number of ABC transporters with high expression)

[d] The level of mutational burden from 0 to 4

[e] The presence of pathogenic mutations from 0 to 2.
